# Effect of interscalene brachial plexus block with dexmedetomidine and ropivacaine on postoperative analgesia in patients undergoing arthroscopic shoulder surgery: a randomized controlled clinical trial

**DOI:** 10.1186/s13063-023-07292-2

**Published:** 2023-06-12

**Authors:** Hengfei Luan, Conghui Hao, Han Li, Xiaobao Zhang, Zhibin Zhao, Pin Zhu

**Affiliations:** 1 Department of Anesthesiology, The First Affiliated Hospital of Kangda College of Nanjing Medical University, The First People’s Hospital of Lianyungang, Lianyungang, 222000 China; 2 Department of Anesthesiology, Jinzhou Medical University, Jinzhou, 121000 China; 3 Department of Anesthesiology, The Affiliated Lianyungang Hospital of Xuzhou Medical University, Lianyungang, 222000 China

**Keywords:** Ropivacaine, Dexmedetomidine, Analgesia, Interscalene brachial plexus block

## Abstract

**Background:**

Dexmedetomidine, a potent and highly selective α2-adrenoreceptor agonist has become a popular adjuvant to local anesthetics. The study was designed to explore the effect of dexmedetomidine added to ropivacaine for interscalene brachial plexus block (IBPB) on postoperative analgesia in patients undergoing arthroscopic shoulder surgery.

**Methods:**

Forty-four adult patients undergoing arthroscopic shoulder surgery were randomly divided into 2 groups. Group R received 0.25% ropivacaine alone, whereas group RD received 0.25% ropivacaine and 0.5 μg/kg dexmedetomidine. A total volume of 15 ml was administered for ultrasound-guided IBPB in both groups. Duration of analgesia, visual analog scale (VAS) pain score, frequency of PCA pressed, first time of PCA pressed, sufentanil consumption, and patient satisfaction with analgesia quality were recorded.

**Results:**

Compared with group R, the duration of analgesia was prolonged (8.25±1.76 vs. 11.55±2.41 h; *P*<0.05), the VAS pain scores were decreased at 8 and 10 h postoperatively (3 (2–3) *vs*. 0 (0–0) and 2 (2–3) *vs*. 0 (0–2.25), respectively; *P*<0.05), the frequencies of PCA pressed were decreased at 4–8 and 8–12 h time intervals (0 (0–0.25) *vs*. 0 (0–0) and 5 (1.75–6) *vs*. 0 (0–2), respectively; *P*<0.05), the time of first PCA pressed was prolonged (9.27±1.85 *vs*. 12.98±2.35 h; P<0.05), the total 24h sufentanil consumption was reduced (108.72±15.92 *vs*. 94.65±12.47 μg; *P*<0.05 ) and patient satisfaction score was also improved (3 (3–4) *vs*. 4 (4–5); *P*<0.05) in group RD.

**Conclusion:**

We concluded that adding 0.5 μg/kg dexmedetomidine to 0.25% ropivacaine for IBPB provided better postoperative analgesia, decreased the sufentanil consumption and improved the patient’s satisfaction in patients undergoing arthroscopic shoulder surgery.

## Introduction

Arthroscopic shoulder surgery is acommon orthopedic procedure performed to treat different shoulder pathologies. Due to the significant advancements in arthroscopic techniques, arthroscopic shoulder surgery is being characterized as a “minimally invasive” procedure[1]. However, it is often associated with moderate to severe postoperative pain that may have a negative influence on patients’ satisfaction and rehabilitation and potentially increase the length of hospital stay[2].

Postoperative pain after arthroscopic shoulder surgery has been managed with the use of patient-controlled intravenous analgesia (PCIA), but the opioids most frequently used in PCIA are associated with adverse effects such as nausea and vomiting, respiratory depression, pruritus, urinary retention, and constipation[3, 4]. In view of this consideration, investigating a multimodal analgesia strategy, which can achieve successful pain management while minimizing opioid usage is recommended.

The brachial plexus provides sensory and motor innervations for the entire upper extremity. Therefore, the interscalene brachial plexus block (IBPB) can provide superior analgesic efficacy and be considered as the gold standard for pain management after arthroscopic shoulder surgery[5]. However, one obvious disadvantage of single-shot IBPB is the short duration of analgesia, which can be resolved by adding various adjuvants to local anesthetics (midazolam, clonidine, tramadol, dexamethasone, and fentanyl)[6, 7].

Dexmedetomidine, a highly selective and potent α2-adrenoceptor agonist, has shown sedative, anxiolytic, analgesic, anti-hypertensive, and sympatholytic properties[8]. Recent clinical trials have demonstrated that adding dexmedetomidine to ropivacaine for an intercostal nerve block or femoral nerve block could provide superior postoperative pain control to ropivacaine alone[9, 10]. On the basis of adding dexmedetomidine to ropivacaine could prolong the duration of analgesia, we hypothesized that adding dexmedetomidine to ropivacaine for IBPB could enhance the analgesic efficacy of ropivacaine. Therefore, we designed a prospective, double-blinded, randomized study to assess the analgesic effect of dexmedetomidine added to ropivacaine for IBPB in patients undergoing arthroscopic shoulder surgery.

## Materials and methods

### Patients

This study was approved by the Hospital Ethics Committee of the First People’s Hospital of Lianyungang (KY-20210423006) and written consent was obtained after informing the participants about the nature, scope, and risks related to the study. The study was also registered with the Chinese Clinical Trials Registry (ChiCTR2100046470). Patients of either sex, with Society of Anesthesiologists (ASA) I or II, between 18 and 65 years of age scheduled for elective arthroscopic shoulder surgery undergoing general anesthesia were eligible. The exclusion criteria were as follows: refusal to receive IBPB, body mass index >30 kg/m^2^, history of severe cardiovascular and respiratory disease, renal or hepatic failure, uncontrolled diabetes, allergy to any of the study drugs, and contraindications to brachial plexus block (coagulopathy or local infection).

### Study design and randomization

A statistician who was not involved in the study conducted the randomization of patients into group R (ropivacaine administration) or group DR (dexmedetomidine and ropivacaine administration) on a 1:1 ratio using a computer-generated random number table. The information regarding the group assignment was placed in an opaque sealed envelope. After the patient entering into the operation room and prior to the induction of anesthesia, the numbered envelope was opened by an anesthesiology nurse, and the card inside determined into which group the patient was placed.

In group R, patients received IBPB using 0.25% ropivacaine 15 ml. In group RD, patients received IBPB using 0.25% ropivacaine and 0.5 μg/kg dexmedetomidine 15 ml. The anesthetic solutions for IBPB were prepared by an anesthesiology nurse who was not involved in the study. The anesthesiologist performing the block and observing the patient was blinded to the treatment group. Data collection was done by the same anesthesiologist who was unaware of the group allocation.

### Procedure of anesthesia

None of the patients were premedicated. After entering the operation room, the patients received routine electrocardiogram (ECG), pulse oxygen saturation (SpO_2_), blood pressure (BP), heart rate (HR), and bispectral index (BIS) monitoring. A 20-gauge cannula was inserted into the dorsum of the patient’s hand and connected to a T-connector for drug administration; Ringer lactate was infused at a rate of 4–6 ml/min. General anesthesia was standardized for all patients in both groups. Patients were preoxygenated with 100% oxygen for 3 min, followed by sufentanil 0.5 μg/kg, propofol 2 mg/kg was intravenously administered, and cisatracurium 0.2 mg/kg was given to facilitate tracheal intubation. Anesthesia was maintained with sevoflurane/O_2_/air mixture to keep BIS values between 40 and 60, muscle relaxation was provided using IV cisatracurium. All surgical interventions were performed by the same surgical team.

### Procedure of IBPB

**Fig. 1:**
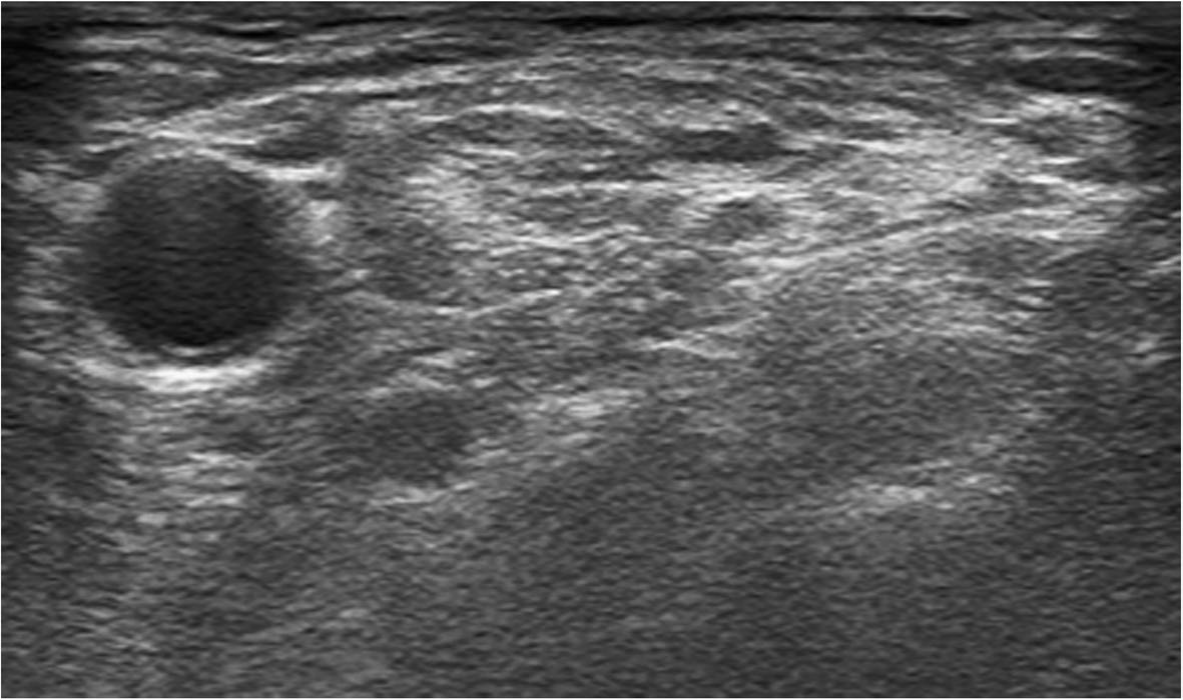
Ultrasound image of interscalene brachial plexus block. ICA, internal carotid artery; ASM, anterior scalene muscle; MSM, middle scalene muscle; BP, brachial plexus; N, needle

The patient was in the supine position with the head slightly turned away from the operative side. The skin was prepared using an antiseptic solution, and the transducer was wrapped in a sterile cover. A 6-13-MHz high-frequency linear probe of the ultrasound (Philips CX50, Philips Ultrasound, Inc., Bothell, WA, USA) was used to identify the C5-C6-C7 nerve roots of the brachial plexus. Then a 21G*100mm insulated needle (UniPlex NanoLine, Pajunk, Geisingen, Germany) was advanced via the lateral-to-medial approach to target the nerve root, and then ropivacaine alone or ropivacaine with dexmedetomidine was injected around the nerve root. All nerve blocks were performed by experienced anesthesiologists who had performed at least 30 blocks with the research technique before beginning the study. At the end of the nerve block, neostigmine 0.04 mg/kg and atropine 0.02 mg/kg were given to reverse the residual neuromuscular block. The patients were extubated awake and transferred to the post-anesthesia care unit (PACU) (Fig. 1).

### Postoperative pain control

All patients received intravenous sufentanil with an intravenous patient-controlled analgesia (PCA) system at the end of surgery. The mode of PCA was a bolus of sufentanil 0.05 μg/kg, a lockout time of 15 min, and a continuous infusion of sufentanil 0.04 μg/kg/h (total regimen 2 μg/kg/100 ml). The patients were taught to push the button of the PCA system to receive a bolus of sufentanil each time pain occurred. If the visual analog scale (VAS) score was ≥ 4, 40 mg parecoxib sodium was injected intravenously as a rescue analgesic.

### Studied variables

Duration of analgesia was defined as the time interval between the completion of IBPB and the time when the patient complained of shoulder pain, and was noted every 30 min since the completion of the IBPB for 4 h.

The VAS (0–10) pain score (VAS; where 0 = no pain and 10 = worst imaginable pain) was assessed at 2, 4, 6, 8, 10, 12, and 24 h after surgery.

Frequency of PCA pressed was recorded at T0–4, T4–8, T8–12, T12–16, T16–20, and T20–24 h postoperatively. The first time PCA was pressed and the total 24h sufentanil consumption were also recorded.

Patient satisfaction with analgesia quality 24 h post-surgery (number rating scale, NRS 1–5; 1, very dissatisfied; 2, dissatisfied; 3, slightly dissatisfied; 4, quite satisfied; 5, completely satisfied) was recorded.

The primary outcome measure in this study was the duration of analgesia. The secondary outcome measures included VAS pain score, frequency of PCA pressed, first time of PCA pressed, total 24h sufentanil consumption, and patient satisfaction with analgesia quality.

### Statistical analysis

The sample size was calculated on the basis of a pilot study taking a mean value of 8.0 h and SD 1.78 h for the duration of postoperative analgesia in 10 patients who received IBPB with ropivacaine. A 20% difference in the duration of the postoperative analgesia was considered a clinically relevant difference. For a 2-group *t* test with α= 0.05, β=0.2, and 2-sided significance level, we required 21 patients in each group. A total of 50 patients were recruited in the study to compensate for possible dropouts.

Statistic tests were performed using SPSS 16.0 for windows (SPSS 16, Chicago, IL, USA). Continuous numerical data were expressed as mean and standard deviation or median and interquartile range. Categorical data were expressed as frequencies and percentages. Normally distributed numerical data between groups were analyzed using the independent 2-sample *t*-test. Skewed data between groups were analyzed using the Mann–Whitney *U*-test. Categorical variables were analyzed using Fisher’s exact test or Pearson’s chi-square test as applicable. All tests were two-tailed. *P* < 0.05 was considered statistically significant.

## Results

**Fig. 2:**
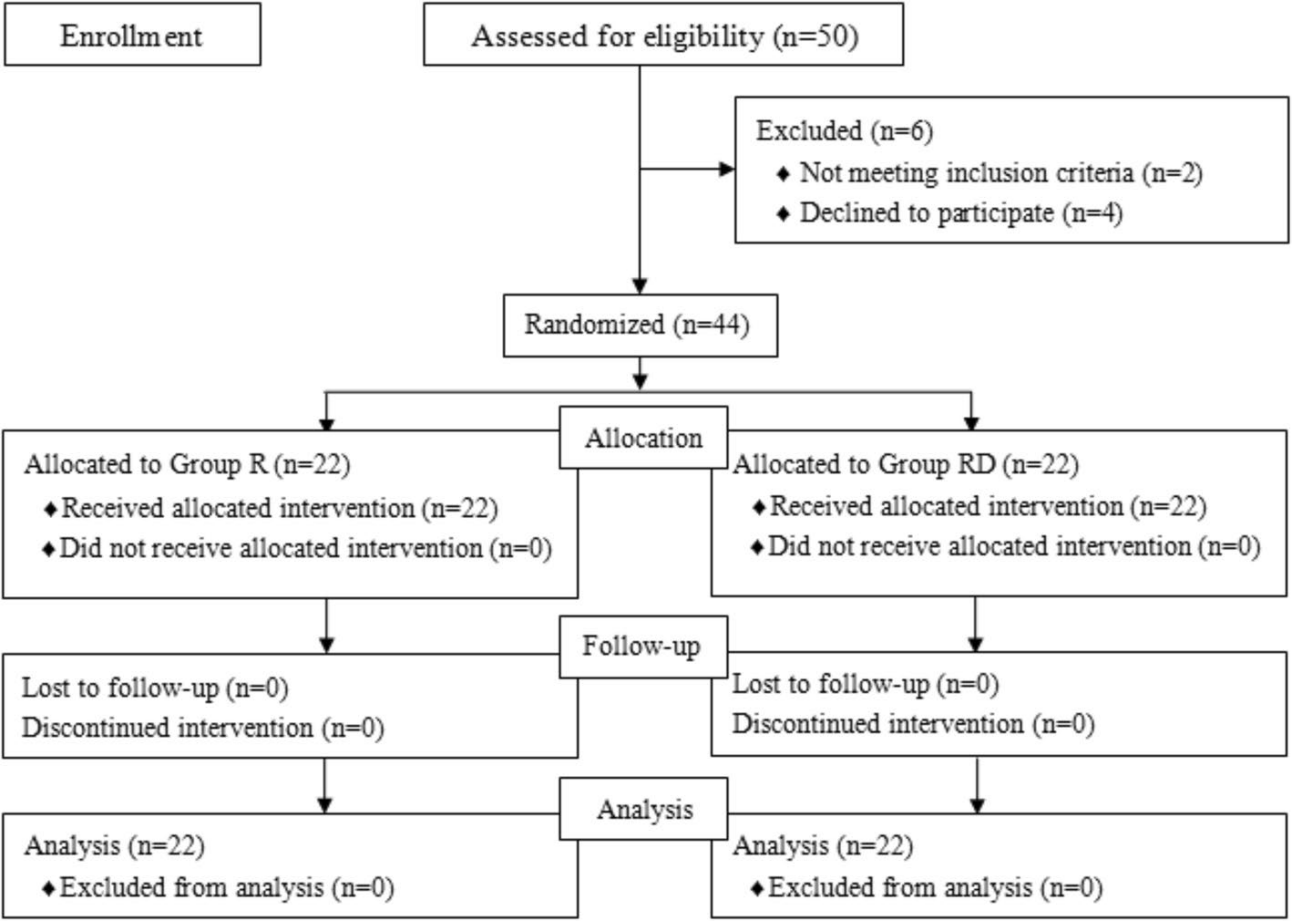
Study flow chart

Among the 50 patients who were eligible for the study, 4 patients refused to participate in the study, and 2 patients received open surgery. Of the remaining 44 patients, 22 patients were randomized to group R, and 22 patients were randomized to group RD (Fig. 2).

There were no differences between groups with respect to demographic data and the operation time (Table 1).

**Table 1:** Comparison of demographics and operation time between the two groups

Demographics	Group R (*n*=22)	Group RD (*n*=22)	*P* value
Age (years)	49.8±9.8	48.9±9.4	0.755
Gender(M/F) (*n*/*n*)	10/12	13/9	0.365
Height (cm)	167.5±7.6	168.4±8.0	0.702
Weight (kg)	71.3±6.6	72.5±6.3	0.548
ASA (I/II) (*n*)	13/9	10/12	0.365
Operation time (min)	108.6±17.8	112.7±22.6	0.509

Data are expressed as mean ± SD or the number of patients

*R* Ropivacaine, *RD* Ropivacaine with dexmedetomidine, *ASA* American Society of Anesthesiologists

The VAS pain scores increased gradually after surgery, due to the analgesic effect of the IBPB fading gradually. The VAS pain scores were significantly lower in group RD at 8 and 10 h postoperatively compared with those in group R. However, there were no significant differences in pain scores between the two groups at 2, 4, 6, 12, and 24 h time points (Table 2).

**Table 2:** Comparison of VAS between the two groups

VAS	Group R (*n*=22)	Group RD (*n*=22)	*P* value
2h	0 (0–0)	0 (0–0)	-
4h	0 (0–0)	0 (0–0)	-
6h	0 (0–0)	0 (0–0)	-
8h	3 (2–3)	0 (0–0)*	<0.001
10h	2 (2–3)	0 (0–2.25)*	0.004
12h	2 (2–3)	2 (0–3)	0.632
24h	1 (1–2)	1 (1–2)	0.760

Data are expressed as median (IQR)

*VAS* Visual analog scale, *R* Ropivacaine, *RD* Ropivacaine with dexmedetomidine

The frequencies of PCA pressed were less in group RD than that in group R at 4–8 and 8–12 h time intervals (*P*<0.05). However, there were no significant differences in frequencies of PCA pressed between the two groups at 0–4, 12–16, 16–20, and 20–24 h time intervals (*P*<0.05) (Table 3).

**Table 3:** Comparison of frequency of PCA pressed between the two groups at different time intervals

Frequency of PCA pressed	Group R (*n*=22)	Group RD (*n*=22)	*P* value
0–4h	0 (0–0)	0 (0–0)	-
4–8h	0 (0–0.25)	0 (0–0)*	0.019
8–12h	5 (1.75–6)	0 (0–2)*	<0.001
12–16h	4 (3–4.25)	3 (2–4)	0.116
16–20h	2 (1.75–3)	2 (1–2.25)	0.673
20–24h	1 (0.75–1.25)	1 (0–1)	0.502

Data is expressed as median (interquartile range) and mean ± SD. *p <0.05

*PCA*, patient-controlled analgesia; *R*, ropivacaine; *RD*, ropivacaine with dexmedetomidine

Compared with group R, the duration of analgesia and the first time PCA was pressed were longer than that in group RD (*P*<0.05). Meanwhile, the total 24h sufentanil consumption in group R was more than that in group RD (*P*<0.05). Patient satisfaction score was also higher in group RD (*P*<0.05) (Table 4).

**Table 4:** Comparison of postoperative variables between the two groups

Postoperative variables	Group R (*n*=22)	Group RD (*n*=22)	*P* value
Duration of analgesia (h)	8.25±1.76	11.55±2.41^*^	<0.001
First time PCA was pressed	9.27±1.85	12.98±2.35^*^	<0.001
Total 24h sufentanil consumption	108.72±15.92	94.65± 12.47^*^	<0.001
Patient satisfaction score	3 (3–4)	4 (4–5)^*^	0.002

Data is expressed as mean ± SD or median (interquartile range), ^*^p<0.05

*R* Ropivacaine; *RD* Ropivacaine with dexmedetomidine

## Discussion

In this prospective, randomized, controlled study, we found that the addition of dexmedetomidine to ropivacaine for IBPB significantly prolonged the duration of postoperative analgesia, prolonged the first time of PCA pressed, reduced the consumption of sufentanil and improved the patient’s satisfaction in patients undergoing arthroscopic shoulder surgery.

The interscalene brachial plexus block is a commonly used regional anesthesia technique, it has been considered as the standard treatment for pain management after shoulder surgery. Ultrasound technology has aided anesthetists in depositing local anesthetics in precise proximity to targeted peripheral nerves, so there is no need to use a large volume of local anesthetics for IBPB. Meanwhile, Studies have demonstrated that local anesthetic volumes of 20 ml or more may associate with a high incidence of hemidiaphragmatic paresis after interscalene brachial plexus block, although it can be resolved by decreasing local anesthetic volume to 5 to 10 ml, this may result in a clinically significant reduction in the duration and potency of perioperative analgesia and may also lead to a high risk of block failure[11–13]. Thus we used 15 ml of 0.25% ropivacaine for IBPB in our study; this is also consistent with the does and concentrations of ropivacaine reported in other centers[14, 15].

The relatively short duration of analgesia is a major limitation of single-shot IBPB. While the catheter-based technique provides sustained pain management during the perioperative period, this technique can present challenges related to patient management, catheter dislocation, and the potential for increased catheter infection risk[16]. Theoretically, Increasing the local anesthetic concentration or volume would affect nerve block duration, However, no changes in mean sensory nerve block duration were reported in participants who received peroneal nerve block with a fixed dose of 10 mg of ropivacaine dissolved in either 2.5, 5, 10, 15, or 20 ml of 0.9% saline and no effect of increasing the volume of ropivacaine 0.2% from 5 to 30 mL on sensory sciatic nerve blocks duration in healthy volunteers received the sciatic nerve[17, 18]. To date, the administration of local anesthetic adjuncts is an attractive and technically simple strategy to potentially extend the benefits of peripheral nerve blockade.

Dexmedetomidine, a highly selective α2 adrenoreceptor agonist, is currently the most widely used additive drug in regional anesthesia. Previous studies have shown that dexmedetomidine can effectively prolong the IBPB analgesic duration and reduce opioid consumption without prolonging motor blockade when administrated intravenously[19, 20]. Meanwhile, a large collection of studies have demonstrated its safety as an anesthetic adjunct when administrated locally. In animal models of spinal anesthesia and sciatic nerve block, dexmedetomidine did not show toxicity and was potentially neuroprotective when combined with lidocaine and bupivacaine[21, 22]. In human trials of peripheral nerve blocks, dexmedetomidine accelerated the onset time of sensory-motor block and prolonged the durations of sensory-motor block and analgesia[23, 24]. However, the local administration of dexmedetomidine is still an off‐label use. Yu et al. reported that 0.5% ropivacaine caused significant sciatic nerve injury in diabetic rats that was greatly potentiated by high-dose dexmedetomidine higher than that used in clinical practice.[25] So it is very important to fully understand the possible adverse events before using dexmedetomidine as a local anesthetic adjuvant to nerve blocks.

In our study, the results showed that compared with 0.25% ropivacaine alone, adding 0.5 μg/kg dexmedetomidine to 0.25% ropivacaine for IBPB prolonged the duration of analgesia approximately 3.5 h on average (8.25 *vs.* 11.55). Postoperative VAS pain scores were comparable in both groups except at 8 and 10 h postoperatively, when the VAS pain scores were lower in group RD compared with group R. Our result was consistent with the result showed by Bharti et al., which indicated that addition of dexmedetomidine to ropivacaine-lidocaine prolonged the duration of supraclavicular brachial plexus block about 5 h (12 *vs*. 17) and also reduced VAS pain scores at 8 and 10 h postoperatively[26].

Multiple basic science studies have demonstrated the effects of perineural dexmedetomidine to be peripheral and not due to systemic analgesia or other α_2_-adrenoceptor effects[27]. But the exact mechanism of action of perineural dexmedetomidine is still unclear, one possible mechanism is the vasoconstriction mediated by acting vascular α-2 adrenoceptors around the site of injection, which delays the absorption of local anesthetic and prolongs the efficacy of local anesthetic[28]. Another possible mechanism is the inhibition of peripheral nerve activity by blocking an activity-dependent cation current (the Ih current), which prevents the returning of nerve from a hyperpolarized state to a resting membrane potential state for subsequent firing[29]. Further studies are still required to explore the exact mechanism.

Patient-controlled analgesia (PCA) is one of the well-established methods for providing postoperative analgesia, a key component for implementing multimodal analgesia. In our study, we used PCA and IBPB as the postoperative analgesic method for arthroscopic shoulder surgery. In fact, no patient required rescue analgesic, which demonstrated our analgesia strategy was successful. Meanwhile, our results showed that dexmedetomidine added to ropivacaine for IBPB prolonged the first time of PCA pressed, decreased the frequencies of PCA pressed at 4–8 and 8–12 h time intervals, reduced the total 24h sufentanil consumption, and improved the patient’s satisfaction. Our results were consistent with a previous study conducted by Yang et al., which concluded that the transversus abdominis plane block reduced morphine consumption in the first 24 h following renal transplantation, and the addition of dexmedetomidine provided a more effective analgesic effect [30].

## Limitations

The present study does have some limitations. First, the study was conducted at a single center and the sample size was relatively small, so the conclusions can not be generalized. Further studies at multiple centers are required to generalize the results. Second, the assessment of VAS pain scores is expected to last for 24 h, especially at the 10h and 12h postoperatively time points, the patient may fall asleep, so the assessment may disrupt the patients’ sleep and impair the patients’ postoperative recovery. Third, it is a standard practice in our center to use 15 ml of 0.25% ropivacaine for IBPB, additional studies are required to research different dosages and concentrations of local anesthetics for IBPB.

## Conclusion

Dexmedetomidine (0.5 μg/kg) added to ropivacaine (0.25%) for interscalene brachial plexus block significantly prolonged the duration of postoperative analgesia, prolonged the first time of PCA pressed, reduced the consumption of sufentanil and improved the patient’s satisfaction in patients undergoing arthroscopic shoulder surgery.

## Data Availability

The datasets generated during and/or analyzed during the current study are available from the corresponding author on reasonable request.
